# Trends and prediction of incidence and mortality burden of larynx cancer in China and the US: a systematic analysis of the Global Burden of Disease Study 2021

**DOI:** 10.3389/fonc.2025.1552514

**Published:** 2025-06-18

**Authors:** Weimin Xu, Hui Gan, Yuhua Ye

**Affiliations:** ^1^ Department of Otolaryngology Head and Neck Surgery, Wuhan Fourth Hospital, Wuhan, China; ^2^ Department of Dermatology, Zhongnan Hospital of Wuhan University, Wuhan, China; ^3^ Department of Otolaryngology, Wuhan Children’s Hospital, Tongji Medical College, Huazhong University of Science and Technology, Wuhan, China

**Keywords:** larynx cancer, global burden of disease, China, the United States, prediction

## Abstract

**Objectives:**

This study aimed to compare and predict the disease burden of larynx cancer in China and the United States (US) using data from the Global Burden of Disease Study 2021 (GBD2021).

**Methods:**

We used the data from GBD2021 to systematically analyze and compare the incidence and mortality of laryngeal cancer in China and the United States, and used the Autoregressive Integrated Moving Average (ARIMA) model to predict the trends in the next 10 years.

**Results:**

In 2021, China had 38,904.86 (95% UI: 30,369.67–49,486.18) larynx cancer cases and 19,799.45 (95% UI: 15,579.57–25,023.24) deaths, reflecting a 2.52-fold and 53.8% increase from 1990. In the US, there were 16,371.45 (95% UI: 15,509.40–17,060.58) cases and 4,620.32 (95% UI: 4,339.97–4,835.98) deaths, showing a 33.3% and 10.04% increase from 1990. Projections indicate a decline in age-standardized incidence and mortality rates in China, while the US is expected to see a slight decline in incidence but continued significant reductions in mortality.

**Conclusions:**

China has a significantly higher number of larynx cancer cases compared to the US, with a higher incidence rate. However, mortality rates are relatively similar. Larynx cancer will remain a notable disease burden in both countries over the next decade.

## Introduction

Larynx cancer poses a significant global public health issue with high morbidity and mortality (1, 2). In 2019, laryngeal cancer accounted for 209,149 (95% UI: 1193876 to 224,620) incidence cases and 123,356 (114,941 to 132,798) deaths globally (3). Incidence and mortality rates vary widely, with the highest incidence in Central Europe and the highest mortality in the Caribbean (3). From 1990 to 2019, the global age-standardized incidence rate decreased by -17.9% (95% UI: -10.8% to -24.0%), the mortality rate decreased by -31.6% (-25.7% to -36.8%) and the DALY rate decreased by -34.2% (-28.1% to -39.6%) (3).

China and the United States, as major economies, both experience substantial burdens from laryngeal cancer. In 2015, China reported approximately 25,300 new cases and 13,700 deaths using data from cancer registries (4). The age-standardized incidence rate in China was 1.18 per 100,000 population, below the global average (4, 5). Although the incidence in China has been rising, mortality rates have been declining (6). The age-standardized mortality rate of larynx cancer in China decreased by 0.9% (95% CI: -1.1 to -0.6) and 2.2% (95% CI -2.8 to -1.7) in males and females, respectively from 1990 to 2019 (6).

In the US, larynx cancer represents a relatively small proportion of overall cancer cases, accounting for about 0.67% of new cancer diagnoses and 0.62% of cancer deaths in 2020 (7). In 2021, there were 12,620 new cases of Larynx tumors and approximately 3,770 deaths in the United States, with the rate in men being significantly higher than in women (7). The incidence and mortality rates for larynx cancer have been on a steady decline since the 1980s, primarily due to decreased smoking rates and improvements in chemotherapy and radiotherapy techniques (8, 9). Nonetheless, disparities in incidence and mortality among different racial and ethnic groups persist (10).

The incidence and mortality of laryngeal cancer change with socioeconomic development. Abdel R. Omran’s research shows that with the improvement of the socioeconomic level of the population, the improvement of public health conditions and the extension of life expectancy, infectious diseases will gradually transform into chronic diseases (11). In addition, differences in lifestyle, socioeconomic status, education level and access to health care may also lead to differences in exposure to laryngeal cancer risk factors in different countries, as well as differences in early diagnosis and treatment (12). Differences in these factors help explain the epidemiological trends, characteristics and overall burden of laryngeal cancer in the two countries.

Key risk factors for larynx cancer include tobacco smoking, alcohol consumption, exposure to asbestos and sulfuric acid, and HPV infection (13–15). Smoking is the predominant risk factor, responsible for roughly 80% of cases in developed nations (16). The risk increases with the duration and intensity of smoking but decreases following cessation (17). Alcohol also significantly raises the risk, especially when combined with smoking (18).

As the world’s largest developing country and developed country respectively, comparing the disease burden of laryngeal cancer between China and the United States has representative significance. The significant differences between the two countries in economic development, healthcare levels, and lifestyles may lead to different epidemiological characteristics of laryngeal cancer. Through comparison, potential factors influencing the incidence and mortality of laryngeal cancer can be revealed, providing a basis for formulating targeted prevention and control strategies. However, there has been limited systematic comparison of the disease’s incidence and mortality trends between China and the US. Such analyses could offer insights into causative factors and aid in developing public health prevention and control strategies. Additionally, predictions on the future burden of larynx cancer are scant, which are crucial for healthcare planning and resource allocation.

This study aims to systematically compare the incidence and mortality trends of larynx cancer in China and the US from 1990 to 2021, utilizing the newly published data from GBD 2021. Additionally, we also employed statistical modeling to predict the future burden of larynx cancer over the next ten years. The results are intended to guide public health policies and interventions for larynx cancer control in both nations.

## Materials and methods

### Study data

Data on the incidence, mortality, and risk factors of laryngeal cancer were obtained from the Global Health Data Exchange (GHDx) query tool (http://ghdx.healthdata.org/gbd-results-tool), which is part of the Global Burden of Disease (GBD) study. This ongoing global collaboration compiles epidemiological data to provide a comparative assessment of health loss from 369 diseases in 204 countries and territories (19). The GBD uses a standardized methodology to estimate disease burden and risk factors globally. The process for deriving risk factor data involves comprehensive literature reviews, meticulous data processing, and advanced statistical modeling. The GBD calculates population attributable fractions for each risk factor by integrating estimates of risk factor exposure with the relative risks of disease outcomes linked to these exposures. Our study specifically utilized the GBD 2021 results tool to extract data on incidence, mortality, and risk factors for laryngeal cancer, including estimated annual percentage change (EAPC) for both China and the US from 1990 to 2021. The data used in this study were anonymized before its use and reviewed by institutional review board of Wuhan Fourth Hospital. All methods were carried out in accordance with relevant guidelines and regulations.

### Data analysis

A secondary descriptive analysis of the burdens of larynx cancer in China and US between 1990 and 2021 was carried out, and the findings were further investigated in different age groups. Bayesian meta-regression with DisMod-MR 2.1 was used as the primary method to estimate each condition. A generalized linear model was used to compute the EAPC of incidence and death cases. Uncertainty intervals (UIs) were defined as the 2.5th and 97.5th values of the posterior distributions. All code is freely available at GHDx.

We developed Autoregressive Integrated Moving Average (ARIMA) models to forecast the age-standardized incidence rates and age-standardized mortality rates of laryngeal cancer in China and the USA. ARIMA is a widely used forecasting technique that combines autoregression and moving average methods to predict future values of time series data and has been used in the past on GBD data (20). The analysis utilized R (Version 2024.12.0 + 467) with the ‘forecast’ package (version 8.24.0).The auto. arima () function from the ‘forecast’ package was employed to automatically select the optimal ARIMA (p,d,q) model specification for each time series. This function determines the necessary order of differencing (d, D) for stationarity and selects the autoregressive (p, P) and moving average (q, Q) orders by minimizing the corrected Akaike Information Criterion (AICc). The auto.arima() procedure includes internal diagnostic checks to ensure the adequacy of the selected models. Training set error measures were calculated to evaluate the in-sample fit of the models and are reported in the Results section. Confidence intervals (80% and 95%) for the ARIMA predictions were also generated. More details of this model were described elsewhere (21).

All data management, statistical analyses and the figures were accomplished by R (version 4.3.1) and R-studio (version 2023.09.1) with “tidyverse”, “ggplot2” and “forecast” packages, et al.

## Results

### Trends in the incidence and mortality of larynx cancer in China and the United States

From 1990 to 2021, the incidence of larynx cancer in China surged by 152% from 15434.15 cases (95% UI:12624.19-18174.01) to 38904.86 cases (95% UI:30369.67-49486.18), with an estimated annual percentage change (EAPC) of 1.52 (95% UI:0.87-2.35). In the United States, the incidence rose by 33.3% from 12275.38 cases (95% UI: 11852.10-12580.97) in 1990 to 16371.45 cases (95% UI: 15509.40-17060.58) in 2021, with an EAPC of 0.33% (95% UI: 0.29-0.38). By 2021, China’s incidence was 1.80 times higher than that of the US (**Table 1**).

**Table 1:** Incidence and death cases of larynx cancer in 1990 and 2021 in USA and China.

Location	Incidence cases, 95%UI			Number of deaths, 95% UI		
	1990	2021	EAPC (%)	1990	2021	EAPC (%)
China	15434.15(12624.19-18174.01)	38904.86(30369.67-49486.18)	1.52(0.87-2.35)	12869.79(10565.15-15142.78)	19799.45(15579.57-25023.24)	0.54(0.15-1.03)
USA	12275.38(11852.10-12580.97)	16371.45(15509.40-17060.58)	0.33(0.29-0.38)	4198.67(4024.92-4304.45)	4620.32(4339.97-4835.98)	0.10(0.06-0.14)

UI, Uncertainty Interval; EAPC, Estimated annual percentage changes.

Mortality from larynx cancer in China increased by 53.8% from 12869.79 cases (95% UI: 10565.15-15142.78) in 1990 to 19799.45 cases (95% UI: 15579.57-25023.24) in 2021, with an EAPC of 0.54% (95% UI: 0.15-1.03). In the US, mortality increased by 10.04% from 4198.67 cases (95% UI: 4024.92-4304.45) in 1990 to 4620.32 cases (95% UI: 4339.97-4835.98) in 2021, with an EAPC of 0.10% (95% UI: 0.06-0.14). In 2021, the mortality cases in China were 3.28 times higher than in the US (**Table 1**).

**Figure 1:**
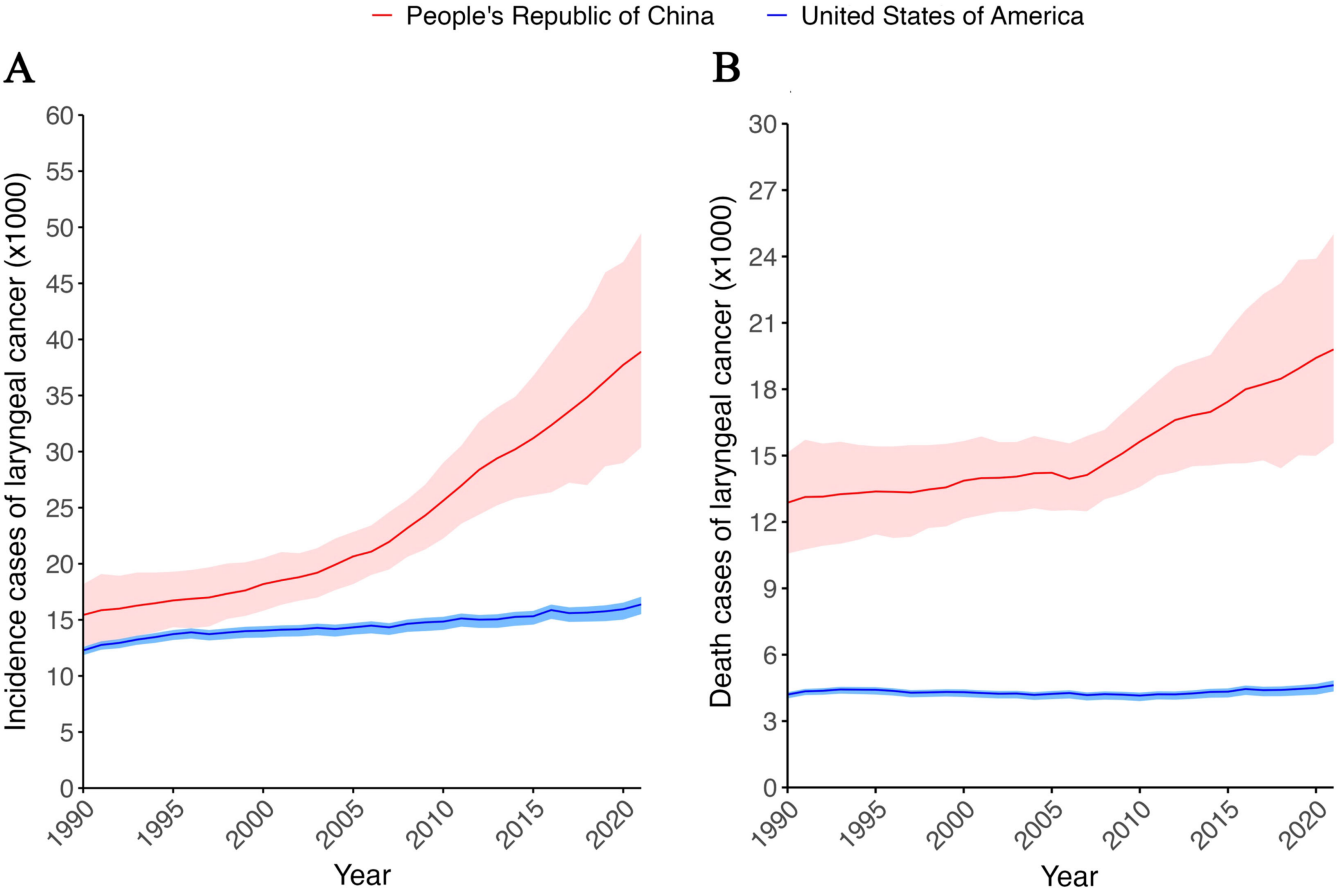
Incidence and death cases of larynx cancer from1990 to 2021 in China and USA. **(A)** Incidence cases of larynx cancer from1990 to 2021 in China and USA. **(B)** Death cases of larynx cancer from1990 to 2021 in China and USA. Red line and ribbon indicate data of China. Blue line and ribbon indicate data of USA.

Over the past 30 years, both the incidence and mortality of larynx cancer have consistently been higher in China compared to the US, with China showing a particularly rapid increase in incidence and mortality after 2005 (**Figure 1**). In contrast to China, the incidence and mortality rates of laryngeal cancer in the United States have been relatively stable over the past 30 years (**Figure 1**).

### Trends in the age-standardized incidence and mortality rates of laryngeal cancer in China and the United States

**Figure 2:**
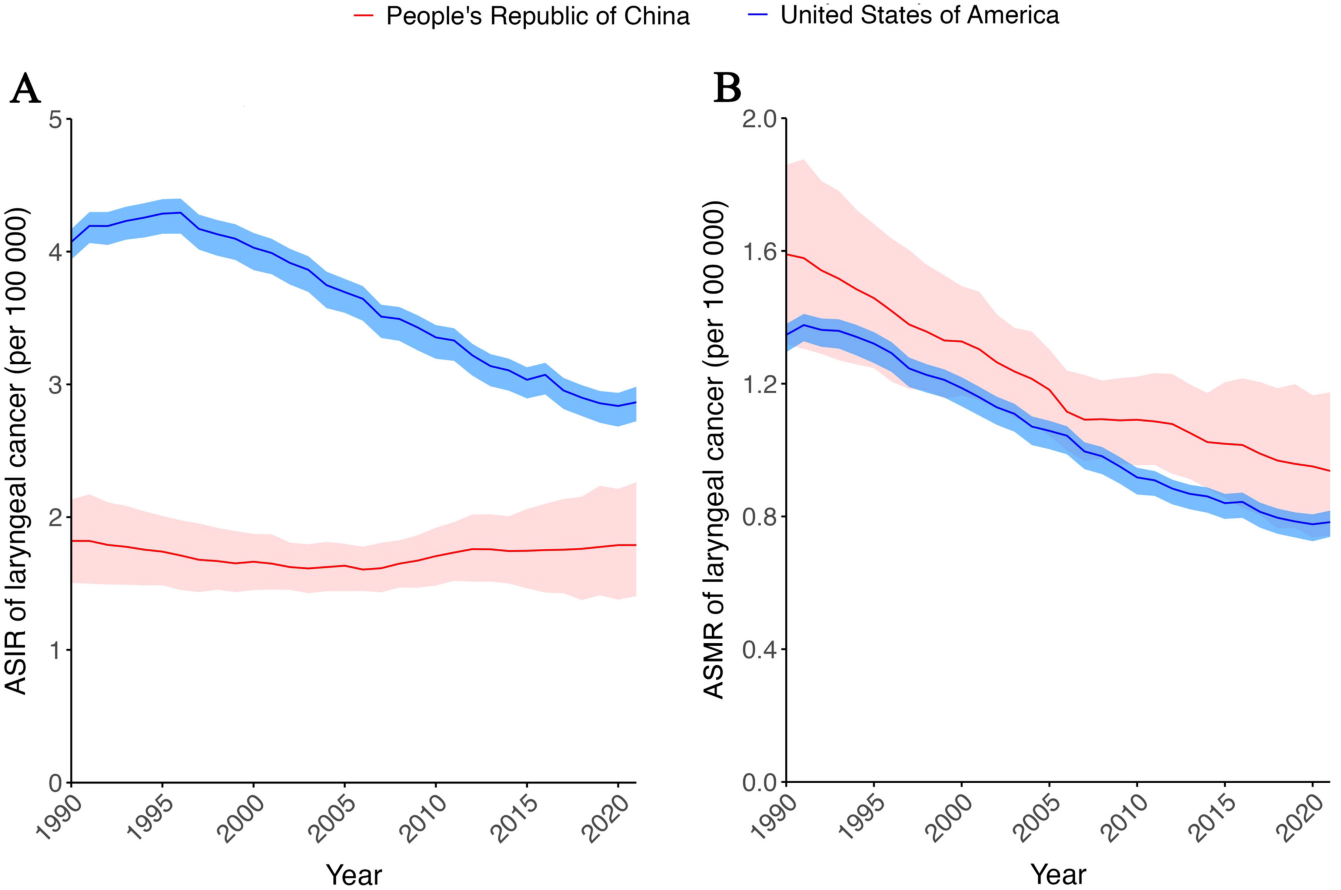
Age-standardized incidence and mortality rate of larynx cancer from 1990 to 2021 in China and USA. **(A)** Age-standardized incidence rate (ASIR) of larynx cancer from 1990 to 2021 in China and USA. **(B)** Age-standardized mortality rate (ASMR) of larynx cancer from1990 to 2021 in China and USA. Red line and ribbon indicate data of China. Blue line and ribbon indicate data of USA.

From the aforementioned results, it can be concluded that both the incidence and mortality of laryngeal cancer have been higher in China than in the United States in absolute numbers over the past 30 years, but considering the larger population size of China, a comparison of age-standardized rates (ASR) is warranted. From a trend perspective, the age-standardized incidence rate (ASIR) of laryngeal cancer in China has shown a slight decline over the past 30 years (**Figure 2A**). Specifically, from 1990 to 2006, the ASIR of laryngeal cancer in China exhibited a very modest downward trend, while after that, it showed a slow upward trend. However, by 2021, the ASIR of laryngeal cancer remained slightly lower than that in 1990 ((1.79 per 100 000 (95% CI: 1.40 - 2.26) vs 1.82 per 100 000 (95% CI: (1.50 - 2.13)) (**Figure 2A**, **Supplementary Table S1**). In contrast, during the same period, the ASIR of laryngeal cancer in the United States showed a significant downward trend. More precisely, the ASIR of laryngeal cancer in the United States demonstrated a marked increase before 1996, followed by a decline. After 2018, the growth trend slowed down and even exhibited a very slight increase (**Figure 2A**, **Supplementary Table S1**). Overall, over the past 30 years, the ASIR of laryngeal cancer in China has been significantly lower than that in the United States, but the gap has been gradually narrowing.

As for the age-standardized mortality rate (ASMR) of laryngeal cancer, both China and the United States have shown a significant downward trend over the past 30 years. However, the ASMR in China has always been higher than that in the United States. Specifically, the ASMR of laryngeal cancer in China showed a rapid decline before 2006, but the rate of decline slowed thereafter. In contrast, ASMR for laryngeal cancer in the United States continued to increase until 1990, peaking in 1991 after just one year of increase, followed by a clear downward trend. However, since 2019, it has changed little (**Figure 2B**, **Supplementary Table S1**).

### ASIR, age-standardized incidence rate. ASMR, age-standardized mortality rate.Age-specific incidence and mortality rates of laryngeal cancer in China and the United States

**Figure 3:**
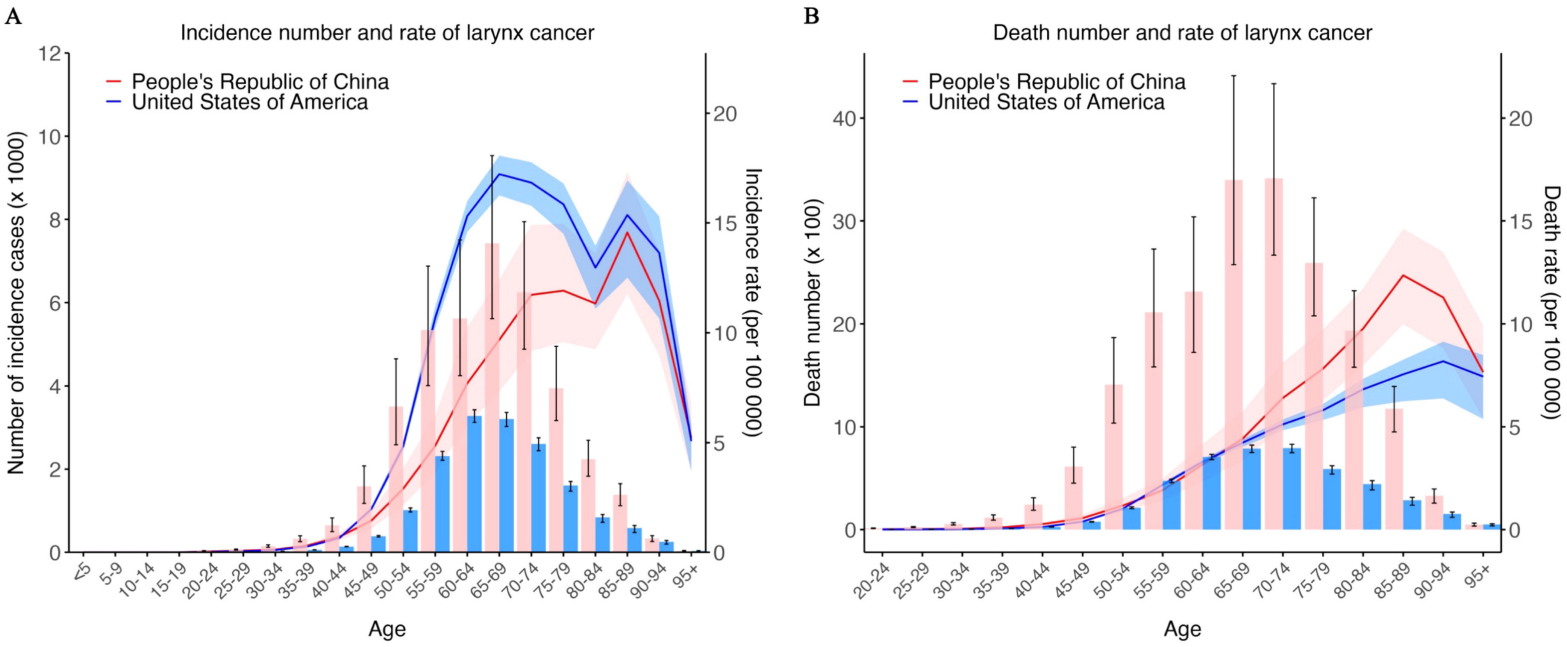
Incidence and death number and rate of larynx cancer in different age group in 2021. **(A)** Incidence number (left) and rate (right) of larynx cancer in different age group in 2021. **(B)** Death number (left) and rate (right) of larynx cancer in different age group in 2021. Red bar, line and ribbon indicate data of China. Blue bar, line and ribbon indicate data of USA. Bar shows the number, line and ribbon show the rate.

Age plays a crucial role in the incidence and mortality of laryngeal cancer. Therefore, we compared the number of cases, incidence rates, number of deaths, and mortality rates of laryngeal cancer across different age groups in China and the United States in 2021 (**Figure 3**). The results show that as age increases, the number of cases and incidence rates of laryngeal cancer gradually rise until reaching a peak value, after which they decline (**Figure 3A**). In China, the number of laryngeal cancer cases begins to decrease after the age of 69, while in the United States, the decline starts after the age of 64. As for the age-standardized incidence rate (ASIR), data from China shows a significant increasing trend with age, peaking in the 85–89 age group, followed by a rapid decline. In contrast, in the United States, the ASIR shows two peaks with age, occurring in the 65–69 age group and the 85–89 age group, with the highest ASIR in the 65–69 age group.

Similarly, for the number of deaths and mortality rates due to laryngeal cancer, both China and the United States show an increase with age until reaching a peak value, followed by a decline (**Figure 3B**). In China, the number of deaths from laryngeal cancer reaches its highest point at the age of 65–69 and then decreases; the same pattern is observed in the United States. There is no significant difference in age-standardized mortality rate (ASMR) for laryngeal cancer between the two countries before the age of 65. However, after the age of 65, China’s laryngeal cancer ASMR is generally higher than that of the United States. The ASMR in China peaks in the 85–89 age group, while in the United States, it peaks in the 90–94 age group.

### Comparison of risk factors for laryngeal cancer mortality in China and the United States

**Figure 4:**
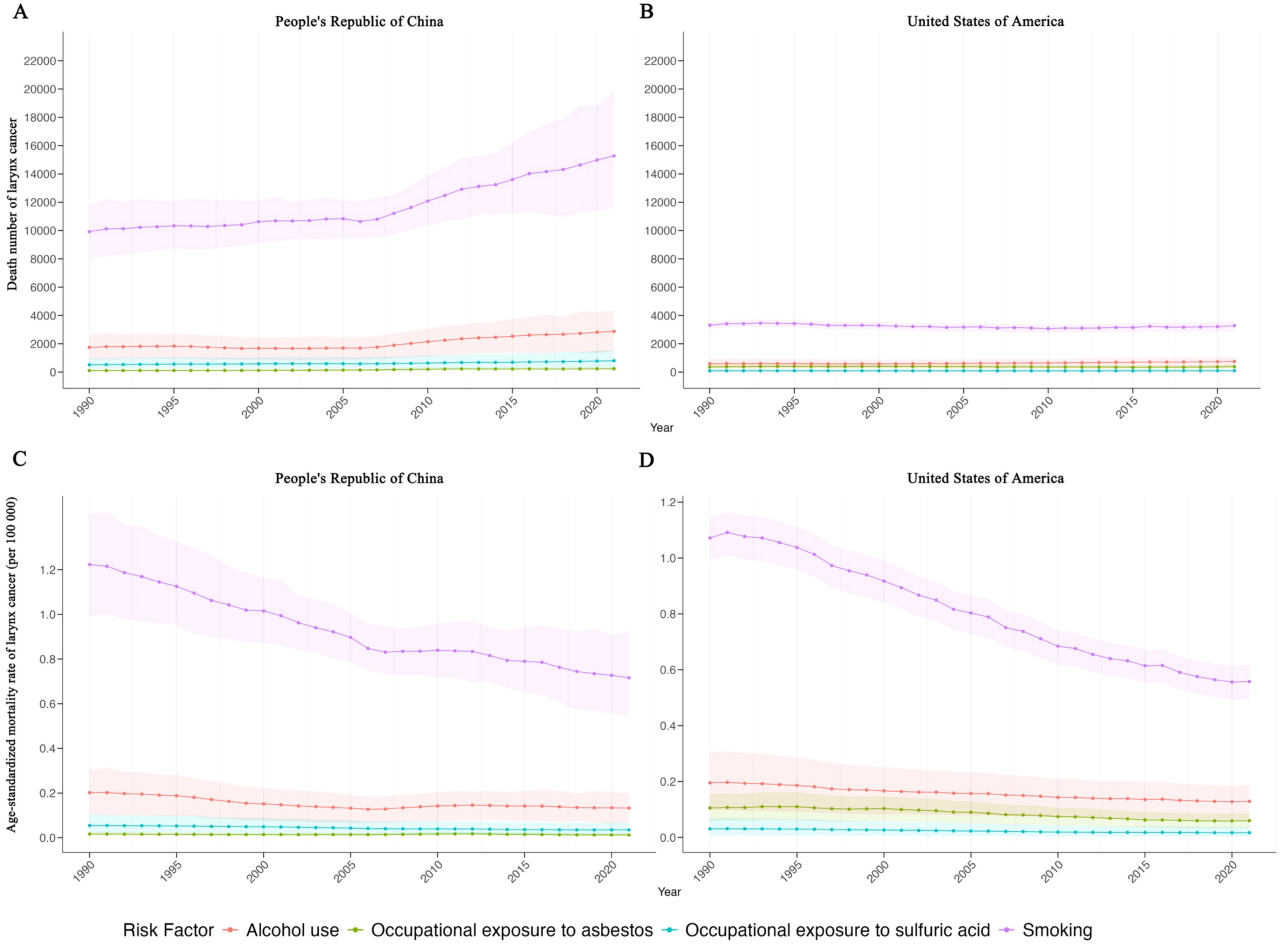
Trend of death number and age-standardized mortality rate of larynx cancer by risk factor in China and USA. **(A)** Trend of death number of larynx cancer by risk factor in China. **(B)** Trend of death number of larynx cancer by risk factor in USA. **(C)** Age-standardized mortality rate of larynx cancer by risk factor in China. **(D)** Age-standardized mortality rate of larynx cancer by risk factor in USA. Purple line and ribbon show data of occupational exposure to sulfuric acid. Orange line and ribbon show data of smoking. Blue line and ribbon show data of occupational exposure to asbestos. Green line and ribbon show data of alcohol use.

Over the last 30 years, the major risk factors for laryngeal cancer mortality have been occupational exposure to sulfuric acid, smoking, occupational exposure to asbestos, and alcohol use (**Figure 4**). In both China and the United States, smoking is the predominant risk factor, followed by alcohol use. In China, occupational exposure to sulfuric acid is the third most significant risk, while occupational exposure to asbestos is the least significant. Conversely, in the US, occupational exposure to asbestos is the third leading risk factor, with occupational exposure to sulfuric acid ranking fourth.

In China, deaths from laryngeal cancer associated with smoking have increased, although the age-standardized mortality rate (ASMR) has decreased (**Figure 4C**). In the US, the number of deaths from laryngeal cancer associated with smoking has slightly fluctuated, but the ASMR has significantly declined (**Figure 4D**). Alcohol use-related deaths in China have slightly increased, with a stable ASMR. In the US, Alcohol use has caused a slight increase in deaths, with a very slight decrease in the ASMR.

Deaths and ASMR due to laryngeal cancer associated with occupational exposure to sulfuric acid and asbestos in China have remained relatively stable over the past 30 years. Likewise, in the US, deaths and ASMR from laryngeal cancer associated with occupational exposure to sulfuric acid have been stable, while those from occupational exposure to asbestos have shown a very slight decline.

### Prediction of laryngeal cancer incidence and mortality in China and the US

**Figure 5:**
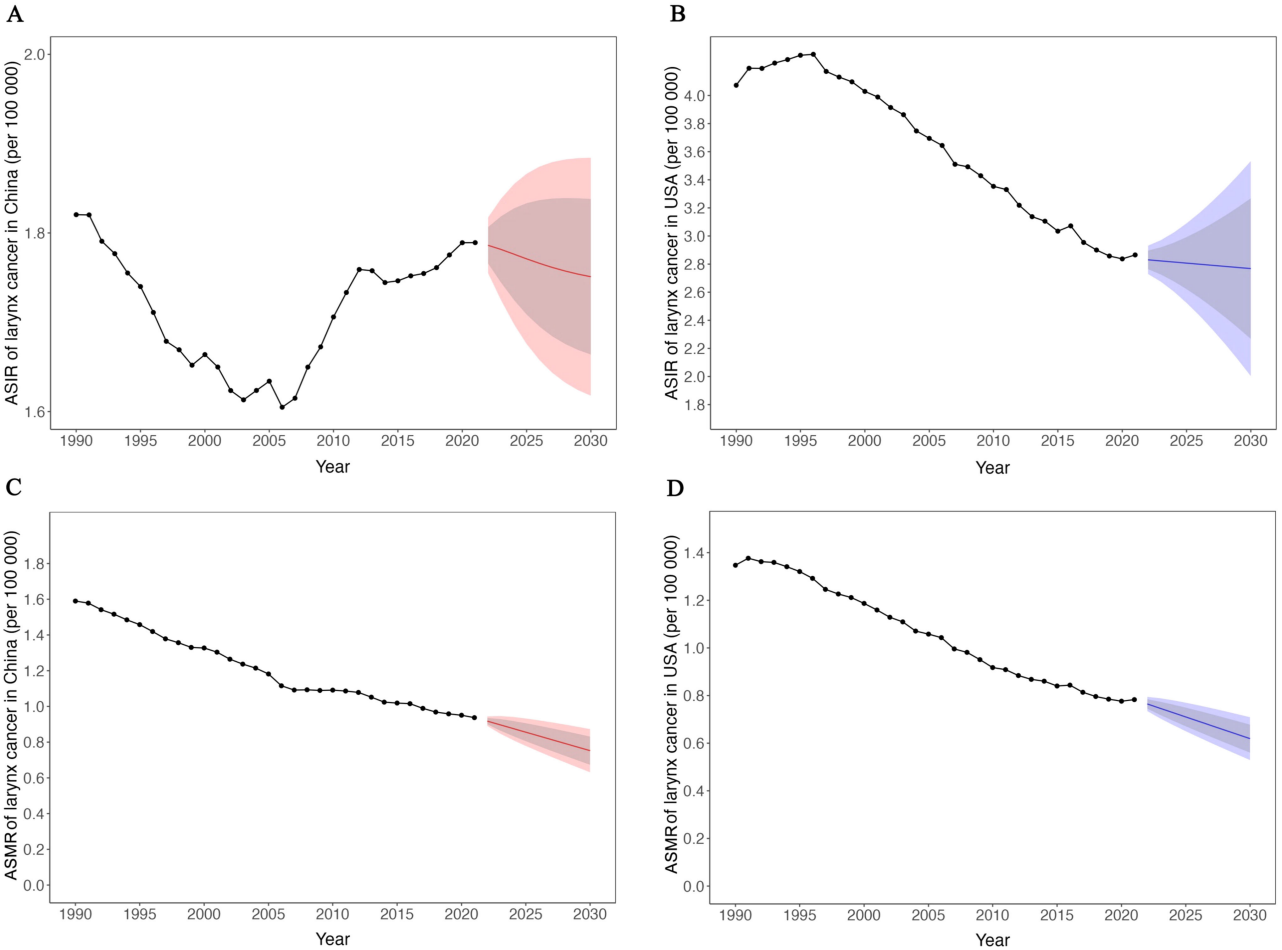
Trend and prediction of age-standardized incidence and mortality rate of larynx cancer in China and USA. **(A)** Trend and prediction of ASIR of larynx cancer in China. **(B)** Trend and prediction of ASIR of larynx cancer in USA. **(C)** Trend and prediction of ASMR of larynx cancer in China. **(D)** Trend and prediction of ASMR of larynx cancer in USA. Red line indicates data of China. Blue line indicates data of USA. Gray ribbon shows the 80% uncertainty interval, red and blue ribbon shows the 95% uncertainty interval.

Using data from the past 30 years, we proceeded to predict the age-standardized incidence and mortality rates of laryngeal cancer in China and the United States. The results show that over the next 10 years, the age-standardized incidence and mortality rates of laryngeal cancer in both countries will maintain their previous trends (**Figure 5**). Specifically, over the next 10 years, ASIR of laryngeal cancer in China is expected to show a moderate downward trend, while in the United States, it will show a slight decrease. As for the ASMR, both China and the United States are projected to experience a significant downward trend.

Based on the prediction data, the ASIR of laryngeal cancer in China in 2032 is estimated at 1.75 per 100,000 (95% UI: 1.61-1.88), compared to 2.75 per 100,000 (95% UI: 1.75-3.75) in the United States, as shown in **Table 2**. Although the ranges suggest a potentially lower rate in China, the overlap in uncertainty intervals indicates that these differences are not statistically conclusive. Overall, over the next 10 years, the age-standardized incidence rate of laryngeal cancer in China will be lower than that in the United States.

**Table 2:** Prediction of age-standardized incidence and mortality rate of larynx cancer from 2020 to 2030 in China and USA.

Year	ASIR of larynx cancer (per 100 000, 95%UI)		ASMR of larynx cancer (per 100 000, 95%UI)	
	China	USA	China	USA
2022	1.79(1.75-1.82)	2.83(2.73-2.93)	0.92(0.89-0.94)	0.76(0.73-0.79)
2027	1.76(1.64-1.88)	2.79(2.34-3.25)	0.81(0.72-0.91)	0.67(0.60-0.75)
2032	1.75 (1.61-1.88)	2.75(1.75-3.75)	0.71(0.58-0.85)	0.58(0.48-0.68)

The prediction for 2032 indicates that the ASMR of laryngeal cancer in China is expected to be 0.71 per 100,000 (95% UI: 0.58-0.85), which is statistically comparable to that of the United States, estimated at 0.58 per 100,000 (95% UI: 0.48-0.68), as both rates have overlapping uncertainty intervals.

## Discussion

A comparative epidemiological study of laryngeal cancer between China and the USA over the past 30 years provides significant insights. It helps identify trends in mortality and incidence rates, which are crucial for developing tailored public health policies and cancer prevention strategies. Additionally, this analysis aids in resource allocation, focusing on research and treatment areas to effectively reduce the disease burden. Predictive modeling from this study also assists in planning and adapting healthcare systems to future challenges. Results revealed that while China experienced higher overall incidence and mortality rates than the US, its age-standardized incidence rate was lower and mortality rates were comparable. The burden of larynx cancer increased with age, peaking and then decreasing in both countries. Key risk factors identified included occupational exposure to smoking, alcohol consumption and occupational exposure to sulfuric acid and asbestos. The forecasts indicate that the incidence and mortality rates of laryngeal cancer in both China and the United States will decrease in the next 10 years.

Despite the forecasts indicating a slowing trend, laryngeal cancer remains a significant health concern in both China and the United States, underscoring the need for ongoing prevention and early detection efforts (22).

The comparison of age-standardized incidence and mortality rates for laryngeal cancer between China and the United States over the past 30 years reveals notable trends. Although China has a significantly higher number of cases, its age-standardized incidence rate (ASIR) remains lower than that of the US, primarily due to its larger population. Nonetheless, the gap in ASIRs is closing, indicating a faster rise in China’s incidence rates. This aligns with previous studies highlighting an increasing burden of laryngeal cancer in China (6, 23). Both countries have seen similar age-standardized mortality rates (ASMR), with a slight downward trend consistent with global decreases in laryngeal cancer mortality (24, 25). This decline may be linked to advancements in treatment, such as improved surgical methods and targeted therapies (26, 27). Variations in ASMRs between China and the US could stem from differences in healthcare access, treatment approaches, and preventive measures (2, 27). In fact, different treatment modes will have a significant impact on the prognosis of laryngeal cancer (28). With the advancement of medical technology in the future, new diagnostic and treatment methods will continue to emerge, which will inevitably have a greater impact on the morbidity and mortality of laryngeal cancer in China and the United States. The Chinese and US healthcare systems differ significantly in funding sources (primarily public vs. private), the role of primary care, patient access pathways, and insurance coverage. These differences may affect the early screening, timeliness of diagnosis, accessibility of treatment, and adherence to follow-up for laryngeal cancer, thus impacting its incidence and mortality (29, 30). Further research is necessary to pinpoint the precise factors behind these differences and to devise targeted interventions to lower the mortality from laryngeal cancer in both nations.

The age-specific incidence and mortality rates of laryngeal cancer in China and the United States follow a pattern of increase until a peak, then decline, particularly prevalent in older age groups (7, 9). This phenomenon, often termed an ‘age-related cohort effect’ or reflecting a ‘real downturn’ in risk among the very elderly in some populations, has also been observed in other epidemiological studies across different regions, though the peak age can vary (31). Such a trajectory strongly suggests that the risk of laryngeal cancer escalates with age due to the cumulative and prolonged exposure to well-established risk factors like smoking and alcohol consumption (18, 32). Notably, the persistently higher mortality rates observed in China post-age 65, when compared to some high-income countries, may indeed be attributable to disparities in healthcare access, the effectiveness of early detection programs, and variations in treatment effectiveness for geriatric patients (33). For instance, research by Wang et al. highlighted that older cancer patients in certain rural areas of China often present with more advanced disease stages at diagnosis, linked to lower awareness of early symptoms and limited access to specialized oncological services (34). These findings collectively underscore the critical need for targeted public health interventions, including enhanced, age-appropriate screening initiatives, improved healthcare infrastructure for geriatric oncology, and equitable access to effective treatments for older populations in China, thereby aiming to mitigate the high mortality burden from laryngeal cancer, a strategy also advocated by global cancer control reports focusing on aging populations (35).

The analysis of laryngeal cancer mortality risk factors in China and the United States identifies smoking and alcohol use as the primary causes of death in both countries, consistent with prior research (8, 36) and corroborated by large-scale case-control study research such as R Talamini et al, who emphasize the particularly strong synergistic effect when these two exposures co-occur, significantly elevating risk beyond their individual contributions (37). In China, despite a decrease age-standardized mortality rates (ASMR), the rise in deaths from laryngeal cancer reflect China’s large population and high smoking rates (38). This situation is further compounded by what Jing Zhang and colleagues describe as ongoing challenges in achieving deep penetration and uniform enforcement of tobacco control policies across all demographics and regions in China (39). Conversely, in the U.S., the significant decrease in ASMR for laryngeal cancer associated with smoking can be directly linked to decades of effective anti-smoking campaigns and comprehensive tobacco control measures (40, 41), as meticulously documented by Levy et al. who modeled the impact of specific interventions like taxation and public smoking bans (42). The stable mortality rates and numbers of deaths from laryngeal cancer associated with sustained alcohol use patterns, and ongoing, albeit potentially reduced, occupational exposures like asbestos and sulfuric acid in both countries (43), suggest these risk factors may have reached a plateau in their overall impact. Nevertheless, this plateau does not mean the problem is over; rather, it highlights that continued, perhaps more targeted efforts—such as stricter enforcement of occupational safety standards and public health initiatives targeting harmful alcohol consumption—remain critical to further reducing laryngeal cancer mortality.

Predictions for laryngeal cancer incidence and mortality over the next decade highlight key trends and disparities between China and the United States. Overall, both countries are expected to experience a decline in age-standardized incidence and mortality rates for laryngeal cancer. This may result from advances in medical technology, particularly new treatment methods and early intervention strategies for laryngeal cancer (44, 45). However, laryngeal cancer will still pose a significant health burden in both countries, particularly in China. To mitigate the rising burden of laryngeal cancer in China, public health strategies should focus on reducing risk factor exposure, enhancing early detection through screening, and improving healthcare access (9, 46). In the U.S., continued efforts are needed to sustain current trends and further decrease incidence and mortality among high-risk groups.

Our study on laryngeal cancer faces several limitations. First, the data from GBD2021 are modeled estimates rather than direct surveillance data, particularly affecting countries with inadequate cancer surveillance. Thus, these estimates come with significant uncertainty, especially for recent years where they rely more on historical trends and covariates. Second, the lack of detailed data prevented subgroup analyses for different laryngeal cancer types, such as anatomical subsites or histological variations, which may differ significantly in clinical behavior. Third, we only evaluated the impact of four risk factors-occupational exposures to sulfuric acid and asbestos, smoking, and alcohol use-on laryngeal cancer mortality. Additional research is necessary to explore other demographic and clinical influences. Fourth, our ten-year predictions for laryngeal cancer burden are based on past trends, which might not accurately reflect future realities due to potential advances in medical technology and changes in risk factor prevalence. Therefore, while this study offers valuable insights into the epidemiological burden of laryngeal cancer in China and the US, there is a clear need for improved cancer surveillance and broader research to achieve a more comprehensive understanding of the disease’s impact.

## Conclusion

In the past 30 years, China experiences a higher and more rapidly increasing incidence and mortality rate compared to the US. Smoking, alcohol consumption, occupational exposure to sulfuric acid and asbestos are key risk factors. This study predicts a certain degree of decline in the age-standardized incidence and mortality rates of laryngeal cancer in both China and the United States over the next decade. However, laryngeal cancer will still pose a significant health burden. To mitigate the burden of laryngeal cancer, China should implement public health measures focusing on risk reduction, early detection, and enhanced healthcare access. In contrast, the US should continue current practices and focus on reducing rates among high-risk groups through targeted interventions.

## Data Availability

The original contributions presented in the study are included in the article/**Supplementary Material**. Further inquiries can be directed to the corresponding author.
