# Supplementary material for: Trends and prediction of incidence and mortality burden of larynx cancer in China and the US: a systematic analysis of the Global Burden of Disease Study 2021
Source: Front Oncol. 2025 Jun 18;15:1552514. doi: 10.3389/fonc.2025.1552514 (PMC12213797; doi:10.3389/fonc.2025.1552514)
Supplement: Supplementary file 1 [file Table1.docx]

Supplement table 1. Age-standardized incidence and death rate of larynx cancer from 1990 to 2021 in China and USA.

|  | Age-standardized incidence rate  (Per 100 0000) | | Age-standardized death rate  (Per 100 0000) | |
| --- | --- | --- | --- | --- |
| Year | China | USA | China | USA |
| 1990 | 1.82(1.50 - 2.13) | 4.07(3.94 - 4.17) | 1.59(1.32 - 1.86) | 1.35(1.29 - 1.38) |
| 1991 | 1.82(1.50 - 2.17) | 4.19(4.06 - 4.30) | 1.58(1.30 - 1.88) | 1.38(1.33 - 1.41) |
| 1992 | 1.79(1.49 - 2.11) | 4.19(4.05 - 4.30) | 1.54(1.29 - 1.81) | 1.36(1.31 - 1.40) |
| 1993 | 1.78(1.49 - 2.09) | 4.23(4.09 - 4.34) | 1.52(1.27 - 1.78) | 1.36(1.31 - 1.39) |
| 1994 | 1.76(1.48 - 2.04) | 4.26(4.11 - 4.36) | 1.48(1.26 - 1.72) | 1.34(1.29 - 1.38) |
| 1995 | 1.74(1.49 - 2.01) | 4.29(4.13 - 4.40) | 1.46(1.25 - 1.68) | 1.32(1.26 - 1.36) |
| 1996 | 1.71(1.45 - 1.98) | 4.29(4.14 - 4.40) | 1.42(1.21 - 1.64) | 1.29(1.24 - 1.33) |
| 1997 | 1.68(1.43 - 1.95) | 4.17(4.02 - 4.28) | 1.38(1.18 - 1.60) | 1.25(1.19 - 1.28) |
| 1998 | 1.67(1.45 - 1.92) | 4.13(3.97 - 4.24) | 1.36(1.18 - 1.56) | 1.23(1.17 - 1.26) |
| 1999 | 1.65(1.44 - 1.90) | 4.10(3.94 - 4.21) | 1.33(1.16 - 1.53) | 1.21(1.16 - 1.24) |
| 2000 | 1.66(1.45 - 1.87) | 4.03(3.86 - 4.14) | 1.33(1.16 - 1.49) | 1.19(1.13 - 1.22) |
| 2001 | 1.65(1.45 - 1.87) | 3.99(3.83 - 4.10) | 1.30(1.15 - 1.48) | 1.16(1.10 - 1.19) |
| 2002 | 1.62(1.45 - 1.81) | 3.91(3.75 - 4.02) | 1.26(1.13 - 1.41) | 1.13(1.08 - 1.16) |
| 2003 | 1.61(1.43 - 1.80) | 3.86(3.70 - 3.97) | 1.24(1.10 - 1.37) | 1.11(1.06 - 1.14) |
| 2004 | 1.62(1.44 - 1.81) | 3.75(3.57 - 3.85) | 1.21(1.08 - 1.36) | 1.07(1.02 - 1.10) |
| 2005 | 1.63(1.44 - 1.80) | 3.69(3.54 - 3.79) | 1.18(1.04 - 1.30) | 1.06(1.00 - 1.09) |
| 2006 | 1.60(1.44 - 1.78) | 3.64(3.48 - 3.74) | 1.12(1.00 - 1.24) | 1.04(0.99 - 1.07) |
| 2007 | 1.61(1.43 - 1.81) | 3.51(3.35 - 3.60) | 1.09(0.97 - 1.23) | 1.00(0.94 - 1.02) |
| 2008 | 1.65(1.47 - 1.83) | 3.49(3.33 - 3.58) | 1.09(0.97 - 1.21) | 0.98(0.93 - 1.01) |
| 2009 | 1.67(1.47 - 1.86) | 3.43(3.26 - 3.52) | 1.09(0.96 - 1.22) | 0.95(0.90 - 0.98) |
| 2010 | 1.71(1.48 - 1.92) | 3.35(3.19 - 3.45) | 1.09(0.95 - 1.22) | 0.92(0.87 - 0.95) |
| 2011 | 1.73(1.52 - 1.96) | 3.33(3.18 - 3.42) | 1.09(0.95 - 1.23) | 0.91(0.86 - 0.94) |
| 2012 | 1.76(1.51 - 2.02) | 3.22(3.07 - 3.31) | 1.08(0.93 - 1.23) | 0.88(0.84 - 0.91) |
| 2013 | 1.76(1.51 - 2.02) | 3.14(2.99 - 3.23) | 1.05(0.91 - 1.20) | 0.87(0.82 - 0.90) |
| 2014 | 1.74(1.50 - 2.01) | 3.11(2.95 - 3.19) | 1.02(0.88 - 1.17) | 0.86(0.81 - 0.89) |
| 2015 | 1.75(1.46 - 2.06) | 3.03(2.89 - 3.13) | 1.02(0.86 - 1.20) | 0.84(0.79 - 0.87) |
| 2016 | 1.75(1.43 - 2.10) | 3.07(2.92 - 3.16) | 1.02(0.83 - 1.22) | 0.84(0.80 - 0.87) |
| 2017 | 1.75(1.43 - 2.14) | 2.95(2.81 - 3.05) | 0.99(0.80 - 1.21) | 0.81(0.77 - 0.84) |
| 2018 | 1.76(1.37 - 2.15) | 2.90(2.76 - 2.99) | 0.97(0.76 - 1.19) | 0.80(0.75 - 0.82) |
| 2019 | 1.78(1.41 - 2.24) | 2.86(2.71 - 2.95) | 0.96(0.77 - 1.20) | 0.78(0.74 - 0.81) |
| 2020 | 1.79(1.38 - 2.21) | 2.84(2.68 - 2.94) | 0.95(0.74 - 1.16) | 0.78(0.73 - 0.81) |
| 2021 | 1.79(1.40 - 2.26) | 2.87(2.72 - 2.98) | 0.94(0.74 - 1.17) | 0.78(0.74 - 0.82) |
